# Health of Spanish centenarians: a cross-sectional study based on electronic health records

**DOI:** 10.1186/s12877-019-1235-7

**Published:** 2019-08-19

**Authors:** Antonio Gimeno-Miguel, Mercedes Clerencia-Sierra, Ignatios Ioakeim, Beatriz Poblador-Plou, Mercedes Aza-Pascual-Salcedo, Francisca González-Rubio, Raquel Rodríguez Herrero, Alexandra Prados-Torres

**Affiliations:** 1 EpiChron Research Group on Chronic Diseases, Aragon Health Sciences Institute (IACS), IIS Aragón, Miguel Servet University Hospital, REDISSEC, Paseo Isabel la Católica, 1-3, 50009 Zaragoza, Spain; 2 Aragon Health Service (SALUD), Miguel Servet University Hospital, Zaragoza, Spain; 3 EpiChron Research Group on Chronic Diseases, IIS Aragón, Zaragoza, Spain; 4 Directorate of Primary Care, SALUD, Zaragoza, Spain; 5 Primary Care Health Centre Delicias-Sur, SALUD, Zaragoza, Spain

**Keywords:** Centenarians, Multimorbidity, Polypharmacy, Healthcare use, Spain

## Abstract

**Background:**

With the number of centenarians increasing exponentially in Spain, a deeper knowledge of their socio-demographic, clinical, and healthcare use characteristics is important to better understand the health profile of the very elderly.

**Methods:**

We conducted a retrospective, cross-sectional observational study in the EpiChron Cohort (Aragón, Spain) aimed at analyzing the socio-demographic, clinical, drug use and healthcare use characteristics of 1680 centenarians during 2011–2015, using data from electronic health records and clinical-administrative databases.

**Results:**

Spanish centenarians (79.1% women) had 101.6 years on average. Approximately 80% of centenarians suffered from multimorbidity, with an average of 4.0 chronic conditions; 50% were exposed to polypharmacy, with an average of 4.8 medications; only 6% of centenarians were free of chronic diseases and only 7% were not on medication. Centenarians presented a cardio-cerebrovascular pattern in which hypertension, heart failure, cerebrovascular disease and dementia were the most frequent conditions. Primary care was the most frequently visited healthcare level (79% of them), followed by medical specialist consultations (23%), hospitalizations (13%), and emergency service use (9%).

**Conclusions:**

Multimorbidity is the rule rather than the exception in Spanish centenarians. Addressing medical care in the very elderly from a holistic geriatric view is critical in order to preserve their health, and avoid the negative effects of polypharmacy.

## Background

The substantial decline in old age mortality of the past decades, together with the large birth cohorts of the early 1900s, has led to more and more people currently living beyond 100 years worldwide [1]. The United Nations, which monitors the global number of centenarians since 1990, stated that this age group will strongly increase from half a million in 2015 to more than 25 million people in 2100 [2]_._ In Spain, their numbers are estimated to increase from 16,460 at present to more than 220,000 in 2066 [3].

Additionally, the rates of multimorbidity (i.e., presence of two or more chronic diseases) [4] and of its associated negative consequences such as higher risk of polypharmacy, disability and functional decline, and inappropriate healthcare use (e.g., hospitalizations by ambulatory care sensitive conditions, readmissions to hospital in a short period of time, overutilization of healthcare services) [5] could be expected to increase in centenarians as the burden of chronic diseases consistently grows with age [6–8]. Notwithstanding, centenarians have been described as a heterogeneous population group in terms of their morbidity profiles [9, 10] and health service use patterns [11], which might be explained in part by the dissimilar populations and methodologies used in the existing literature [12–14].

Some studies suggest that the burden of chronic conditions in centenarians is low, with low lethality rates [10, 15, 16] and low healthcare use [17]. Richmond et al. [16] conducted a study based on structured health history questionnaires and reported a relatively low prevalence of chronic conditions, with many centenarians escaping cardiovascular disease, osteoporosis, dementia, respiratory illness, cancer, anxiety and depression. However, the positive results reported by Australian centenarians might be related in part to the fact that this study was based on self-perceived health information from a convenience sample with various recruitment methods. By contrast, the Danish Centenarian Study [9] showed high levels of morbidity, particularly in hypertension (52%), dementia (51%), ischemic heart disease (27%), and strokes (22%); and a study of Tokyoite centenarians [18] found that more than 95% of them had chronic diseases.

The most commonly reported diseases in centenarians vary among studies and countries, including chronic conditions and symptoms such as joint pain (64%), hypertension (40–64%), cataracts/ocular disease (47–71%), arthritis (58%), left ventricular dysfunction (54%), heart disease (29–31%), osteoporosis (28%), gastrointestinal disease (21%), depression (18%), and cerebrovascular disease (16%), and also conditions such as fractures (47%) and dizziness (44%) [16, 18, 19].

Understanding the health profile and health care needs of the very elderly and generally frail, such as centenarians, becomes especially relevant when considering their numbers are increasing exponentially. Although the more methodologically sound research studies reported high prevalence of chronic diseases and multimorbidity in centenarians, no studies on the health status or healthcare use of Spanish centenarians have been published in the English literature. With our data including almost all centenarians in a geographical region that is representative of the whole of Spain we expect to provide sound scientific knowledge to this field of study. This could facilitate the design of specific care models fitting the socio-demographic and clinical characteristics of centenarians, and the identification of potential underlying factors for shorter longevity. The aim of this study was to describe the socio-demographic, clinical and healthcare use characteristics of a cohort of Spanish centenarians using routinely collected data from electronic health records and clinical-administrative databases.

## Methods

### Design and study population

**Fig. 1:**
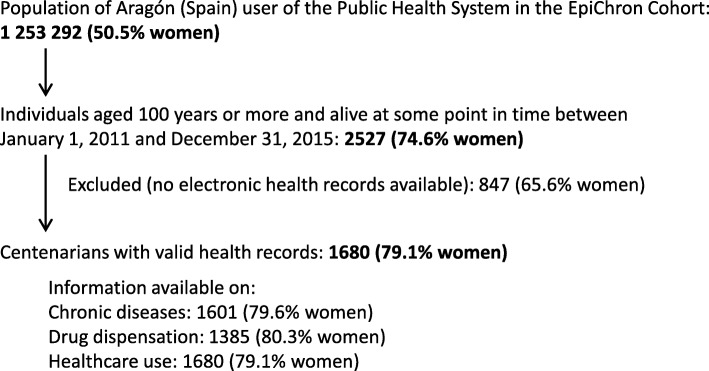
Flowchart for the study population

We conducted a retrospective, cross-sectional observational study in the EpiChron Cohort [20]. This cohort links socio-demographic, clinical, health services use, and drug dispensation information for the public health system users of the Spanish region of Aragón (1.3 million inhabitants). The reference population of the EpiChron Cohort was 1,253,292 individuals at baseline (i.e., January 1, 2011), which represents approximately 98% of total inhabitants. The study population was composed of people from the cohort aged 100 years or more who were alive at some point in time from January 1, 2011 to December 31, 2015. The anonymized data used in the study was obtained from electronic health records and clinical-administrative databases linked at the patient level (i.e., user database, primary, specialist, hospital and emergency care, and pharmacy billing databases). A more detailed description of the cohort profile and of the data sources has been published elsewhere [20]. Only patients with at least one contact with the health system registered in their electronic health records were included in the study (Fig. 1).

The information analyzed included age, sex, area of residence (urban/rural), deprivation index of the area (according to 26 socioeconomic indicators) [21], all diagnoses of chronic diseases, all chronic medications dispensed, use of potentially inappropriate medications according to the updated Beers criteria [22], and healthcare use rates. All the information analyzed corresponded to the last 365 days of follow-up of each participant, which ended with either death of the patient, withdrawal of the system, or on December 31, 2015. Diagnoses were grouped in Expanded Diagnostic Clusters (EDCs) based on the Johns Hopkins ACG® System (version 11.0, The Johns Hopkins University, Baltimore, MD, US). This classification system is useful in multimorbidity studies to count diseases when diagnoses from different sources and codification systems are used (e.g., ICPC-1 codes from primary care and ICD-9-CM codes from hospitals). For the analysis of multimorbidity, defined as the co-occurrence of two or more chronic diseases, we considered all 114 of the EDCs defined as chronic by Salisbury et al. [23]. We defined chronic medications as those with three or more dispensations over the 365-days follow-up period, using the Anatomical-Therapeutic-Chemical (ATC) classification system code at the third level. We calculated the anticholinergic drug scale score [24] and the anticholinergic cognitive burden [25] for each patient based on their medical prescriptions.

### Statistical analysis

We performed a descriptive analysis of socio-demographic, clinical, healthcare use and drug use characteristics of centenarians, by sex. The results were calculated as means and/or frequencies accompanied by their 95% confidence intervals. We used the Kruskal-Wallis test to compare means and the Pearson’s chi-squared test to compare frequencies. Statistical significance was set at *p* < 0.05. We conducted all the analyses in Stata (Version 12.0, StataCorp LLC, College Station, TX, US).

## Results

### Demographics of centenarians

A total of 1680 people (79.1% women) of the EpiChron Cohort were 100 years old or above at some point in time from January 1, 2011 to December 31, 2015 and had valid electronic health records. Their socio-demographic characteristics are shown in Table 1. They had an average age of 101.6 (standard deviation, s.d., 1.84) years, with no differences by sex. The maximum age registered was 111 years in women and 109 years in men. Two women were supercentenarians (i.e., 110 years old or older), and 32 men and 101 female were semi-supercentenarians (i.e., aged 105 years or more). Regarding their place of residence, 57% of centenarians lived in an urban area during the study period. Approximately 30% of them lived in the less deprived areas (i.e., Q1) according to the deprivation index calculated for each basic health area of the region.

**Table 1:** Socio-demographic, clinical and healthcare use characteristics of centenarians, by sex. Means are accompanied by their 95% confidence intervals in brackets

	Men	Women	*P* value
Socio-demographics			
N	351	1329	<0.001
Mean age, years^a^	101.7 (101.5–101.9)	101.6 (101.5–101.7)	n.s.^b^
Urban residence, %	58.7%	56.7%	n.s.
Deprivation index, %^c^			n.s.
Q1	29.1%	31.1%	
Q2	23.7%	22.8%	
Q3	24.0%	20.7%	
Q4	23.1%	25.6%	
Clinical information			
Without chronic diseases, %	7.6%	5.2%	n.s.
Chronic diseases, mean^a^	3.97 (3.65–4.28)	3.97 (3.81–4.12)	n.s.
With multimorbidity, %	78.0%	81.3%	n.s.
Without chronic medications, %	8.1%	6.4%	n.s.
Chronic drugs, mean^a^	4.94 (4.56–5.33)	4.77 (4.58–4.95)	n.s.
With polypharmacy, %^d^	49.8%	49.5%	n.s.
With potentially inappropriate medications, %^a,e^	71.1%	72.3%	n.s.
Potentially inappropriate medications, mean^a^	1.32 (1.19–1.45)	1.36 (1.29–1.43)	n.s.
Anticholinergic Drug Scale score, %			n.s.
0	50.6%	50.2%	
1	28.2%	29.5%	
2	12.4%	12.3%	
≥3	8.8%	8.0%	
Anticholinergic Cognitive Burden score, %			n.s.
0	55.3%	52.3%	
1	26.0%	28.8%	
2	6.2%	8.5%	
≥3	12.5%	10.4%	
Healthcare use			
Users of primary care services, %	76.1%	79.4%	n.s.
Visits to GP^f^, mean^a^	10.6 (9.4–11.7)	10.8 (10.2–11.5)	n.s.
Visits to nurse, mean^a^	9.3 (7.8–10.9)	12.1 (10.9–13.3)	n.s.
Users of specialities, %	24.5%	22.3%	n.s.
Visits to a specialist, mean^a^	2.79 (2.16–3.43)	2.72 (2.44–2.99)	n.s.
Different specialties visited, mean^a^	1.37 (1.23–1.51)	1.47 (1.37–1.57)	n.s.
Users of hospital, %	14.8%	12.1%	n.s.
Total hospital admissions, mean^a^	1.35 (1.17–1.52)	1.34 (1.23–1.45)	n.s.
Scheduled hospital admissions, mean^a^	0.06 (0.00–0.12)	0.12 (0.06–0.17)	n.s.
Length of stay in days, mean^a^	10.0 (7.9–12.0)	11.6 (10.3–13.0)	n.s.
Users of emergency room, %	11.7%	8.3%	n.s.
Visits to emergency room, mean^a^	1.80 (1.40–2.21)	1.45 (1.28–1.62)	n.s.

^a^A non-parametric test was used; ^b^Not statistically significant; ^c^From less (Q1) to most (Q4) deprived administrative health areas; ^d^Defined as five or more drugs dispensed; ^e^According to Beers criteria; ^f^General Practitioner

### Disease burden in centenarians

Centenarians showed an average disease burden of almost 4 chronic conditions with no differences by sex, and approximately 6% of them did not present any chronic condition (Table 1). The prevalence of multimorbidity reached 80% of the centenarian population.

The ten most frequently reported chronic conditions in centenarians were, regardless of their sex, hypertension (56.0%), chronic ulcer of skin (28.1%), cerebrovascular disease (21.5%), congestive heart failure (20.3%), dementia (19.9%), degenerative joint disease (17.1%), varicose veins of lower extremities (14.9%), cataract/aphakia (13.9%), cardiac arrhythmia (11.7%), and diabetes (11.1%; Table 2). Incontinence was present in 49.5% of centenarians. The prevalence of some cardiovascular risk factors, such as hypertension and lipid metabolism disorders, as of other conditions such as chronic ulcer of the skin, dementia, degenerative joint disease, varicose veins, and osteoporosis was higher in women. On the other hand, men showed higher prevalence rates of COPD, other respiratory disorders, gout, and low back pain.

**Table 2:** Prevalence of chronic conditions in men (*n* = 351) and women (*n* = 1329) centenarians. Conditions are listed in descending order of total prevalence, and only those with prevalence equal to or greater than 1% are represented

EDC^a^ code	Chronic condition	Men (%)	Women (%)	*P* value
CAR14/15	Hypertension	46.8	58.4	<0.001
REC03	Chronic ulcer of the skin	22.0	29.7	<0.01
NUR05	Cerebrovascular disease	22.3	21.3	n.s.^b^
CAR05	Congestive heart failure	20.2	20.3	n.s.
NUR24	Dementia	12.8	21.7	<0.001
MUS03	Degenerative joint disease	13.2	18.1	<0.05
GSU08	Varicose veins of lower extremities	8.6	16.5	<0.001
EYE06	Cataract, aphakia	14.7	13.7	n.s.
CAR09	Cardiac arrhythmia	14.1	11.1	n.s.
END06/07/08/09	Diabetes	10.1	11.4	n.s.
EAR08	Deafness, hearing loss	8.6	10.9	n.s.
CAR11	Disorders of lipid metabolism	6.4	11.4	< 0.05
PSY19	Sleep disorders of nonorganic origin	8.9	10.8	n.s.
HEM02	Iron deficiency, other deficiency anemias	11.9	9.7	n.s.
EYE08	Ischemic heart disease or AMI^c^	11.6	9.1	n.s.
PSY09	Glaucoma	8.0	8.9	n.s.
END02	Depression	7.3	8.2	n.s.
CAR03	Osteoporosis	1.8	9.3	<0.001
RES04	Emphysema, chronic bronchitis, COPD^d^	14.7	5.4	<0.001
SKN02	Dermatitis and eczema	8.6	6.1	n.s.
HEM03	Malignant neoplasms	9.7	4.3	<0.001
NUR21	Thrombophlebitis	3.4	5.8	n.s.
GUR04	Neurologic disorders, other	5.8	5.0	n.s.
RES11	Prostatic hypertrophy	24.2	0.0	<0.001
EYE02	Respiratory disorders, other	7.0	4.2	<0.05
MAL01	Visual impairment	5.5	4.6	n.s.
CAR16	Skin/low impact neoplasms	5.5	3.8	n.s.
END04	Cardiovascular disorders, other	3.1	3.9	n.s.
END05	Hypothyroidism	3.1	3.8	n.s.
REN01	Other endocrine disorders	1.8	4.1	n.s.
RHU02	Chronic renal failure	4.9	3.0	n.s.
NUT03	Gout	7.0	2.1	<0.001
ADM02	Obesity	1.5	3.5	n.s.
CAR12	Surgical aftercare	3.4	2.8	n.s.
HEM08	Hematologic disorders, other	4.3	2.4	n.s.
ALL04/05	Asthma	1.5	3.1	n.s.
GAS10	Diverticular disease of colon	2.5	1.9	n.s.
PSY14	Psychosocial disorders of childhood	1.2	2.2	n.s.
PSY01	Anxiety, neuroses	1.2	2.0	n.s.
CAR06	Cardiac valve disorders	1.8	1.8	n.s.
MAL12	Low back pain	3.1	1.0	<0.05
MUS14	Irritable bowel syndrome	0.9	1.3	n.s.
GAS09	Seizure disorder	2.1	1.0	n.s.
NUR07	Utero-vaginal prolapse	0.0	1.4	<0.05
FRE12	Parkinson’s disease	1.5	1.0	n.s.
NUR06	Gastroesophageal reflux	0.6	1.2	n.s.
GAS08	Peripheral neuropathy, neuritis	0.6	1.2	n.s.

^a^From a list of 114 Expanded Diagnostic Clusters; ^b^Not statistically significant; ^c^Acute myocardial infarction; ^d^Chronic obstructive pulmonary disease

### Drug use in centenarians

Centenarians presented a mean treatment burden of 4.8 chronic medications (Table 1), and 49.5% of them received five or more drugs (i.e., polypharmacy). Only 7% of centenarians had no chronic medications dispensed. According to the updated Beers criteria from 2015 [22] and to anticholinergic scores [24], centenarians received on average 1.35 potentially inappropriate medications, 8% of them were treated with high anticholinergic risk drugs, and 11% suffered a high anticholinergic cognitive burden, with no differences by sex.

Gastroprotective drugs (50.9%), antithrombotic agents (40.6%), analgesics and antipyretics (36.0%), diuretics (34.2%), anxiolytics (21.5%), antidepressants (14.4%), hypnotics-sedatives (13.8%), antipsychotics (13.6%), and ACE (angiotensin converting enzyme) inhibitors (13.4%) were the most frequently dispensed chronic drugs in centenarians (Table 3). The dispensation of anxiolytics, hypnotics-sedatives, and opioids, among others, was higher in women compared with men; whereas the latter were prescribed more non-steroidal anti-inflammatory and antirheumatic products, antigout preparations, and inhalants for obstructive airway diseases and adrenergics.

**Table 3:** Prevalence of most dispensed chronic medications in men (*n* = 351) and women (*n* = 1329) centenarians. Drugs are listed in descending order of total prevalence, and only those with prevalence equal to or greater than 1% are represented

ATC^a^ code	Chronic medication	Men (%)	Women (%)	*P* value
A02B	Drugs for peptic ulcer and GERD^b^	53.9	50.2	n.s.^c^
B01A	Antithrombotic agents	43.6	39.9	n.s.
N02B	Other analgesics and antipyretics	34.1	36.4	n.s.
C03C	High-ceiling diuretics	31.5	34.9	n.s.
N05B	Anxiolytics	13.9	23.4	<0.001
N06A	Antidepressants	12.8	14.7	n.s.
N05C	Hypnotics and sedatives	9.9	14.7	<0.05
N05A	Antipsychotics	11.4	14.2	n.s.
C09A	ACE^d^ inhibitors, plain	7.3	14.9	<0.001
B03A	Iron preparations	12.8	10.7	n.s.
C08C	Selective calcium channel blockers with mainly vascular effects	13.9	10.3	n.s.
C05C	Capillary stabilizing agents	4.4	11.4	<0.05
C01D	Vasodilators used in cardiac diseases	8.1	10.3	n.s.
C09C	Angiotensin II receptor blockers, plain	7.0	9.0	n.s.
M02A	Topical products for joint and muscular pain	6.1	9.0	n.s.
C09D	Angiotensin II receptor blockers, combinations	5.9	8.4	n.s.
S01E	Antiglaucoma preparations and miotics	7.7	7.3	n.s.
A06A	drugs for constipation	8.1	6.7	n.s.
M01A	Antiinflammatory and antirheumatic products, non-steroids	10.3	6.1	<0.05
C01A	Cardiac glycosides	8.1	6.6	n.s.
N06B	Psychostimulants, agents used for ADHD^e^ and nootropics	8.8	6.0	n.s.
R05C	Expectorants, excl. Combinations with cough suppressants	8.4	5.5	n.s.
A10B	Blood glucose lowering drugs, excl. Insulins	5.1	5.9	n.s.
V06X	General nutrients	5.5	5.6	n.s.
B03B	Vitamin B12 and folic acid	5.1	5.5	n.s.
C07A	Beta blocking agents	4.4	5.7	n.s.
N02A	Opioids	2.2	6.2	<0.05
G04C	Drugs used in benign prostatic hypertrophy	26.0	0.0	<0.001
C10A	Lipid modifying agents, plain	6.6	4.3	n.s.
C04A	Peripheral vasodilators	5.5	4.1	n.s.
R03A	Adrenergics, inhalants	7.3	3.7	<0.05
R03B	Other drugs for obstructive airway diseases, inhalants	7.3	3.7	<0.05
S01X	Other ophthalmologicals	3.7	4.4	n.s.
C09B	ACE inhibitors, combinations	3.7	4.3	n.s.
D01A	Antifungals for topical use	4.0	4.2	n.s.
C03D	Potassium-sparing agents	5.9	3.6	n.s.
C03E	Diuretics and potassium-sparing agents in combination	1.1	4.6	<0.05
A03F	Propulsives	1.1	4.1	<0.05
R06A	Antihistamines for systemic use	5.5	3.1	n.s.
C01E	Other cardiac preparations	3.7	3.4	n.s.
C03B	Low-ceiling diuretics, excl. Thiazides	2.6	3.6	n.s.
C08D	Selective calcium channel blockers with direct cardiac effects	3.7	2.7	n.s.
A12A	Calcium	0.7	3.3	<0.05
A12B	Potassium	1.8	3.1	n.s.
D07A	Corticosteroids, plain	4.8	2.3	<0.05
M04A	Antigout preparations	5.9	2.0	<0.001
N03A	Antiepileptics	1.8	3.0	n.s.
N07C	Antivertigo preparations	2.9	2.6	n.s.
J01 M	Quinolone antibacterials	4.0	1.9	n.s.
H02A	Corticosteroids for systemic use, plain	3.7	1.7	n.s.
H03A	Thyroid preparations	1.1	2.3	n.s.
J01X	Other antibacterials	2.9	1.4	n.s.
J01C	Beta-lactam antibacterials, penicillins	2.6	1.4	n.s.
N06D	Anti-dementia drugs	1.8	1.6	n.s.
V06Z	General nutrients	2.9	1.4	n.s.
S01A	Antiinfectives	0.7	1.7	n.s.
A10A	Insulins and analogues	1.5	1.4	n.s.
C03A	Low-ceiling diuretics, thiazides	0.7	1.4	n.s.
J01D	Other beta-lactam antibacterials	1.1	1.4	n.s.
R05D	Cough suppressants, excl. Combinations with expectorants	2.3	1.0	n.s.
H03B	Antithyroid preparations	0.7	1.1	n.s.

^a^Anatomical Therapeutic Chemical Classification System; ^b^Gastroesophageal reflux disease; ^c^Not statistically significant; ^d^Angiotensin-converting-enzyme; ^e^Attention deficit hyperactivity disorder

### Healthcare use of centenarians

Primary care was the most frequently visited level of care by centenarians. It was used by 79% of them, with an average of 11 annual visits to the General Practitioner (GP) and to the nurse (Table 1). Specialists were less frequently visited, and only 23% of centenarians received specialized care, with an average of 2.7 visits per year to 1.5 different specialities. The most visited specialists were radiologists (56.6% of the visits to a specialist), ophthalmologists (9.3%), orthopaedists and traumatologists (5.1%), dermatologists (4.4%), cardiologists (3.5%), and endocrinologists, nutritionists and urologists (2.3%), among others. More than 85% of centenarians did not require hospitalization during the study period. Those that used hospital care were admitted 1.3 times per year on average with a mean length of stay of 11 days. Unplanned hospitalizations were much more frequent than scheduled ones and represented 92.5% of the total number of hospitalizations. The ten most prevalent reasons for hospital admission were heart failure (in 12.0% of admissions), femoral neck fractures (10.0%), other respiratory system diseases (9.4%), pneumonia (6.5%), occlusion of cerebral arteries (3.5%), other lung diseases (3.5%), pneumonitis due to solids and liquids (3.3%), septicaemia (2.5%), acute renal failure (2.2%), and other urethral and urinary tract disorders (2.0%). Approximately nine in ten centenarians did not require visiting the emergency room; the rest visited this service 1.5 times per year on average. Sex had no effect on the utilization of any healthcare service.

## Discussion

This study describes the socio-demographic, clinical and healthcare use characteristics of 1680 Spanish centenarians of the EpiChron Cohort between 2011 and 2015 based on electronic health records and clinical-administrative data sources. As far as we know, this is the first study describing the health status of Spanish centenarians in the English literature using register-based health record data from a population-based cohort. As expected, the vast majority of people who reached age 100 were women [26].

Multimorbidity prevalence has been shown to increase as we get older [23, 27]. This concurs with the results from the EpiChron Cohort, where multimorbidity prevalence increased from 12% in the population aged 0–14 years to 16, 47, and 80% in people aged 15–44, 45–64 and ≥ 65 years, respectively [20]. According to this trend, one might have expected multimorbidity prevalence in centenarian populations to be above the 80% observed in the present study. However, our results do not necessarily indicate a lower-than-expected burden of chronic diseases, and could just signify physical and/or environmental difficulties in accessing health care services, or a ‘laissez-faire’ attitude or ageism [28] that could ultimately result in lower diagnosis rates.

Medical literature regarding the prevalence of centenarians without a diagnosis of common chronic conditions is inconsistent. In the Danish Centenarian Study [9] the authors found that only one out of 207 subjects was free of actual diseases or chronic conditions. A Japanese study found it to be less than 3% [18], another study reported that 19% of centenarians did not present chronic diseases [10], and yet another study found that 23% of centenarians reached age 100 with no major chronic diseases [29]. The proportion of disease-free centenarians in our study (6%) is within this range, nonetheless, these figures should be interpreted with caution, since previous studies apply varying methodologies and approach sample recruitment and condition analysis differently. In our study, we used a comprehensive list of more than 100 diseases, in contrast with the lists used by other authors [10, 29]. Additionally, data sources were of great importance when interpreting the results. Electronic health records offered an objective method of patient clinical characterization through the diagnosis of previously confirmed medical conditions by healthcare professionals. Nevertheless, although the public health system in Spain provides universal coverage, the absence of registered clinical information does not necessarily mean an absence of disease. Furthermore, health care service use for diagnostic or therapeutic purposes may have been rejected by a number of centenarians. In the Danish Centenarian Study [9] all participants (including proxies) were visited at their domicile for an interview, and further health information was retrieved from medical files and national health registers.

The most frequent diseases observed in centenarians vary among studies. The high prevalence of cardiovascular diseases found in our study is, however, a common finding, and has also been reported in 72 and 40% of Danish and New England centenarians, respectively [9, 10]. Some of the diseases from the cardio-cerebrovascular disease pattern showed by our centenarians (i.e., hypertension, heart failure, cerebrovascular disease, and cardiac arrhythmia) have also been reported in other studies as the most prevalent in this age group. Hypertension prevalence was similar in Spanish, Danish and Japanese centenarians [9, 18], while heart failure prevalence was lower in our study compared to that reported by Danish and Canadians [9, 17]. Cardiovascular and cerebrovascular diseases are the only frequent conditions in our study that associate high mortality rates [10, 15]. The prevalence of diabetes mellitus in our centenarians (11%) was similar to the 6–10% reported by other authors despite using different methodologies [9, 10, 18]. High dementia prevalence is also a common finding in centenarians [30], especially in women [17], whereas chronic ulcers of the skin are more likely to be a consequence of prolonged limitations in mobility as a result of underlining disorders.

According to our findings, one in two centenarians was polymedicated. The most frequently dispensed medications were similar to those in a Swedish study of community dwelling centenarians [31]. The proportion of individuals in our study with no drug prescriptions (7%) was similar to the 5% found in the Danish Study [9]. A study conducted in the United Kingdom (UK), with an observation period of over twenty years using a Primary Care database of 11,000 individuals who reached the age of 100, reported that 27% of centenarians had no drug prescriptions [32]. The mean burden of treatment in our centenarians, however, was lower, maybe because we only included chronic medication. It is also worth noting that our centenarians were treated with drugs with a low anticholinergic load. Despite all that, the percentage of individuals receiving inappropriate medication according to the Beers criteria was double when comparing with the UK study [32].

Some of the differences between men and women in our results regarding drug dispensation could be reasonably attributed to gender-related morbidities, while others could suggest unequal treatment of specific conditions (i.e., gender inequalities) [33]. For example, the overprescription of certain drugs, such as anxiolytics and hypnotics, was greater in women than in men, even though there were no significant differences in the prevalence of sleep disorders or anxiety/neuroses when stratifying by sex. Similarly, women were more often treated for pain with stronger analgesics, like opioids, when comparing with men.

Healthcare use in centenarians was lower than expected by age regardless of the level of care analyzed. The relatively low burden of chronic diseases and medications in centenarians resulted, as expected, in less intensive use of medical services [34]. These findings could even suggest a more appropriate pattern of healthcare use in centenarians, who mainly resorted to primary care instead of specialized care, and were not frequent users of emergency services. Our results are similar to those found in a previous study based on administrative health data of 1842 Canadian centenarians, who used primary care, hospital care and emergency services in 95, 18 and 26% of cases, respectively [17]. However, these results should be interpreted with caution as they might be explained in part by physical and/or environmental difficulties faced by these patients in accessing health care services.

### Strengths and limitations of the study

The main strength of this study is that it draws on a population-based cohort representative of the Spanish population, although only users of the public health system with available electronic health records were considered for inclusion. As our main limitation, data used in the study was anonymized and centenarians could not be contacted to collect extra variables related to longevity that could have been of interest for the purposes of this study, such as lifestyle habits (e.g., smoking habit, alcohol consumption), level of physical functioning (e.g., activities of daily living), and biological, educational, and socioeconomic (e.g., housing conditions) indicators. Instead, data came from electronic health records and clinical-administrative databases, which, while constituting primary sources of clinical information, were not primarily designed for research purposes and, consequently, could entail some errors during the registration process.

## Conclusions

The utilization of electronic health records allowed for the characterization of the health profile and healthcare use of Spanish centenarians. Multimorbidity seems to be the rule rather than the exception in this population, which mainly presented a cardio-cerebrovascular pattern including hypertension, heart failure, cerebrovascular disease, dementia, and skin ulcers. Addressing medical care in the very elderly from a holistic geriatric view is critical in order to preserve their health, and avoid the negative effects of polypharmacy. Future studies aimed at characterizing the health profile and longevity determinants of centenarians should also take into account genetic, environmental and socioeconomic factors as well as lifestyle habits and physical functionality.

## Data Availability

The datasets generated and analyzed during the current study are not publicly available due to the possibility of compromising individual privacy because of the small number of participants included, but are available from the corresponding author on reasonable request.

## Abbreviations

ACE: angiotensin-converting-enzyme
AMI: acute myocardial infarction
ATC: Anatomical-Therapeutic-Chemical
CEICA: Clinical Research Ethics Committee of Aragón
COPD: chronic obstructive pulmonary disease
EDCs: Expanded Diagnostic Clusters
ER: emergency room
GERD: gastroesophageal reflux disease
GP: General Practitioner
s.d: standard error
